# Exploring Scotopic Microperimetry as an Outcome Measure in Choroideremia

**DOI:** 10.1167/tvst.13.9.29

**Published:** 2024-09-30

**Authors:** Laura J. Taylor, Amandeep S. Josan, Daniel Adeyoju, Jasleen K. Jolly, Robert E. MacLaren

**Affiliations:** 1 Nuffield Laboratory of Ophthalmology, Nuffield Department of Clinical Neurosciences, University of Oxford, Oxford, UK; 2 Oxford Eye Hospital, Oxford University Hospitals NHS Foundation Trust, Oxford, UK; 3 Vision and Eye Research Institute, Anglia Ruskin University, Cambridge, UK

**Keywords:** scotopic microperimetry, microperimetry, choroideremia, outcome measure, fundus-controlled perimetry

## Abstract

**Purpose:**

Choroideremia is an X-linked outer retinal degeneration. Early symptoms include nyctalopia and progressive visual field loss, but visual acuity is preserved until late disease stages. Dark-adapted two-color fundus-controlled perimetry (also known as scotopic microperimetry) has been developed to enable spatial assessment of rod and cone photoreceptor function. This study explores the use of scotopic microperimetry in patients with choroideremia.

**Methods:**

Twenty patients with choroideremia and 21 age-matched healthy controls completed visual acuity and scotopic microperimetry testing, which used the Scotopic Macular Integrity Assessment (S-MAIA) microperimeter. A subset of participants completed repeat scotopic testing to enable Bland–Altman repeatability analyses. Test reliability was assessed using fixation stability, fixation losses, and assessment of the rod-free zones. Pointwise sensitivity, mean sensitivity, and volume sensitivity indices were analyzed.

**Results:**

False positive responses were the main source of poor test reliability, indicated by stimuli responses in the physiological blind spot and lack of rod-free mapping. Scotopic cyan and red sensitivities were significantly reduced in choroideremia participants (*n* = 17) compared to healthy controls (*n* = 16) (*P* < 0.01, Mann–Whitney *U* test). Scotopic cyan sensitivity was statistically lower than scotopic red sensitivity in both healthy controls and choroideremia (*P* < 0.01, Wilcoxon signed rank test). Interpretation of scotopic cyan–red differences should be used with caution due to high test–retest variability.

**Conclusions:**

Scotopic microperimetry could be a useful outcome measure in patients with early choroideremia. Careful selection of test grid design and sensitivity indices is required.

**Translational Relevance:**

Scotopic microperimetry may be a useful outcome measure in clinical trials for patients with early stage choroideremia.

## Introduction

Choroideremia is a progressive X-linked inherited outer retinal degeneration, primarily affecting the retinal pigment epithelium, with subsequent degeneration in the photoreceptors and choroid. The disease is due to loss-of-function mutations in the *CHM* gene that inhibit the Rab escort protein activity that is required to mediate photoreceptor and retinal pigment epithelial cell membrane transport.^1^ Patients with choroideremia present in early teens with nyctalopia and peripheral visual field loss due to impaired rod photoreceptor function. This field loss gradually progresses, causing severe visual impairment typically by the third or fourth decade.^2^ Visual acuity (VA) is preserved until late disease stages; therefore, VA is insensitive to detecting early changes in visual function. This limits its usefulness as a clinical trial outcome measure.^3^^,^^4^

Mesopic microperimetry, also known as fundus-controlled perimetry or simply microperimetry, has been shown to be a robust and repeatable assessment of central retinal sensitivity in many rod–cone degenerations, including choroideremia. As a result, it is now a popular outcome measure in many clinical trials for inherited retinal diseases.^4^^,^^5^ Studies have shown that mesopic microperimetry is a marker of central cone function.^6^^–^^8^

Assessments for localized rod photoreceptor function are more limited. Traditional methods for assessing rod photoreceptor vision (scotopic vision) include dark adaptometry, which assesses the time to adapt at a pre-determined locus.^9^ Global scotopic functional measures, which include full-field stimulus testing, have been shown to be useful in the detection of rod photoreceptor function in choroideremia.^10^ However, due to the spatial insensitivity of the global assessment, it is most useful in patients with very low vision who have no central fixation but do have remaining small peripheral islands of vision.^11^ An additional scotopic rod test is the International Society for Clinical Electrophysiology of Vision (ISCEV) standard flash scotopic full-field electroretinography.^1^ Although this benefits from being purely an objective test, the electroretinography responses suffer from significant floor effects and are often undetectable in patients with rod–cone degenerations, including choroideremia, thus limiting their use as an outcome measure in clinical trials for potential therapies.^12^^,^^13^ Overall, none of these tests provides assessment of spatial variation in rod photoreceptor function across the retina.

Scotopic microperimetry, adapted from mesopic microperimetry, has subsequently been developed to overcome this issue by combining microperimetry central retinal sensitivity testing with dark-adapted two-color perimetry.^14^ Examination is performed in very low lighting conditions (background luminance <0.001 cd/m^2^). The examination experience is nearly identical to standard mesopic microperimetry except that, instead of white stimulus presentations, cyan stimuli (wavelength 505 nm) are used to target rod driven responses, and red stimuli (wavelength 627 nm) are used to elicit mixed rod–cone responses (reference to cyan and red stimuli henceforth will be in the context of scotopic conditions).^7^ The cyan and red stimuli have been calibrated according to the International Commission on Illumination (CIE) 1951 scotopic luminosity function in healthy individuals, where the cyan threshold is approximately 20 decibels (dB) lower than the red threshold. In radiance, the red stimuli, at 0.0 dB, is approximately 20.0 dB brighter than for cyan at 0.0 dB. Therefore, in a healthy retina, the cyan–red difference should be 0.0 dB. A negative cyan–red difference outside of the rod-free fovea suggests reduced cyan response compared to red response and, hence, greater rod dysfunction relative to any cone dysfunction.^14^^,^^15^ A positive cyan–red difference suggests greater cone dysfunction relative to rod dysfunction.^15^^,^^16^ The physiological absence of rod photoreceptors at the fovea is confirmed by the presence of a central cyan scotoma.

Scotopic microperimetry has been shown to be a useful and early marker of visual dysfunction in patients with age-related macular degeneration, macular telangiectasia, and Stargardt disease.^15^^,^^17^^,^^18^ Because early symptoms of choroideremia include nyctalopia, assessing scotopic visual function is a logical approach that should enable detection of subtle changes in central retinal sensitivity, theoretically at earlier disease stages than possible using mesopic microperimetry.

The primary aim of this study was to comprehensively explore the use of scotopic microperimetry in a cohort of patients with choroideremia, as well as healthy controls, to determine the potential for scotopic microperimetry to be used as an outcome measure in future clinical trials. First, test reliability was assessed to determine whether patients with choroideremia could complete the testing reliably. Test sensitivity results were evaluated to determine whether scotopic microperimetry was able to detect reduced scotopic sensitivity. Repeatability analyses enabled identification of the level of sensitivity change required for a clinically significant result that was beyond test–retest variability. The use of the cyan versus red sensitivity differences, a unique feature of two-color scotopic microperimetry, was explored to aid interpretation of reduced rod relative to cone sensitivity in choroideremia. Finally, the study explored structure–function correlations to gain an understanding of how sensitivity changes relate to structural markers and to provide insight into whether scotopic microperimetry sensitivity changes have potential to serve as an earlier marker of change prior to retinal structure degeneration.

## Methods

Healthy controls and patients with a clinical diagnosis of choroideremia were assessed as part of the Visual Function in Retinal Degeneration study (UK research ethical approval reference nos. 20/WM/0283, ISRCTN24016133).^19^ Additional patients with choroideremia were assessed as part of the screening process but prior to the recruitment into a gene therapy clinical trial (UK research ethical approval reference no. 15/LO/1379). All data were collected in accordance with the tenets of the Declaration of Helsinki. Any patients with VA less than 6/60 were excluded. Participants with co-pathologies such as diabetic retinopathy, glaucoma, or other ocular disease or history of ocular surgery were also excluded from the study.

### Scotopic Microperimetry

Scotopic microperimetry was undertaken using the iCare Scotopic Macular Integrity Assessment (S-MAIA) microperimeter (Centervue S.p.A., Vigonza, Italy). The S-MAIA has an integrated scanning laser ophthalmoscope to enable high image quality for real-time eye-tracking correction (at a sampling speed of 25 Hz) to ensure assessment of the same retinal loci, to a high degree of accuracy across different tests. A near-infrared super luminescent diode camera was used to view the fundus (out to 36°) throughout testing.^20^

Each participant underwent 20 minutes of dark adaptation (light level <1.0 lux) prior to testing without any formal pupil dilation.^21^^–^^23^ The S-MAIA default 37-point scotopic radial grid was used, with a background luminance of <0.001 cd/m^2^, a 4.0-2.0 dB bracketing threshold staircase strategy, and Goldmann size III (0.43°) stimulus, with stimuli of various intensities (between 0.00064 and 2.545 cd/m^2^) spanning a 36.0-dB dynamic range. Cyan stimuli testing was performed first followed by red stimuli testing. A 1° circular red target was used for fixation. If this was not seen at the minimum brightness, the luminance was increased until the fixation target was visible to the participant. Before testing, each participant received an explanation about the device and instructions for performing the test. Throughout testing, the examiner monitored the testing performance and periodically gave supportive verbal prompts and encouragement to each participant to help them maintain concentration and improve test reliability. A break was given between each stimuli color testing regime; however, this was not formally timed. Only one eye from each participant was tested. Most participants were experienced with microperimetry testing and so no formal learning test was undertaken. Only participants who were completely microperimetry naïve completed a learning test. This was a custom cyan stimuli test performed under scotopic conditions, prior to dark adaption, on the testing eye only. It included eight rectilinear threshold test points, four central and four more peripherally located.

A subset of participants underwent repeat scotopic microperimetry testing (on the same eye and on the same day), following a short break from the initial test. These participants completed all the cyan stimuli tests prior to beginning the red stimuli testing so as not to impair the dark-adapted rod sensitivities. No additional learning tests were undertaken during repeat testing.

### Mesopic Microperimetry

A further subset of participants also completed mesopic microperimetry on the same study eye using the same S-MAIA microperimeter device. This was performed in a darkened room (light level < 1.0 lux) without any formal dark adaption or pupil dilation.^21^^,^^24^ The standard 10-2 test grid was used, again with 4.0-2.0 dB bracketing threshold strategy, and a Goldmann size III stimulus of various intensities (0–318 cd/m^2^) was presented on a mesopic background (1.27 cd/m^2^). The stimulus dynamic range was 36.0 dB. The same 1°-diameter red circle was used as the fixation target. Prior to testing, subjects were instructed about the test and what they were required to do. During testing, performance was monitored, and participants were again prompted and verbally encouraged to ensure test reliability. The non-tested eye was occluded throughout.

### Retinal Imaging

Fundus autofluorescence 55° images were taken (on choroideremia participants only) using the SPECTRALIS HRA-OCT confocal scanning laser ophthalmoscope (Heidelberg Engineering, Heidelberg, Germany) following pupil dilation. The residual island area of hyperfluorescence was traced using the area measurement tool available in the Heidelberg Eye Explorer software, which calculates the defined area in square millimeters and was previously described by Aylward et al.^3^

### Microperimetry Metrics

#### Reliability Indices

##### Fixation

Fixation stability outputs from the S-MAIA include P1 and P2, which correspond to the percentage of fixation points falling inside 1° and 2° radii of the preferred retinal locus, respectively.^20^ This enables a numerical classification of the fixation stability as described by Fujii et al.^25^ where, for fixation to be classified as stable, P1 must include >75% of fixation points. For a relatively unstable fixation, classification P1 must include <75% and P2 must include >75% of fixation points. An unstable fixation classification occurs when <75% of fixation points are within P2.^25^ Bivariate contour ellipse area (BCEA) indicates the area and orientation of a two-dimensional ellipse encompassing a given proportion (either 95% or 63%) of the fixation points. The smaller the BCEA value, the better the fixation stability.^26^ False-positive responses (termed fixation losses) were measured throughout testing, with suprathreshold stimuli presented to the physiological blind spot and expressed as the percentage seen.

##### Rod-Free Zone Mapping

Identification of the rod-free zone was used as an additional indicator of response reliability. The standard 37-point radial grid has one test point centered on the physiological fovea. Because this is within the rod-free zone, it was assumed that cyan sensitivity at this point should be either 0.0 dB or very low (a low value possibly indicating a very bright cyan stimulus seen by the central cone population). A high central cyan threshold, indicating a lack of the rod-free zone mapping, suggests the results are likely to be unreliable.

#### Sensitivity Indices

Microperimetry threshold sensitivities are provided in decibels, with a 1.0-dB change corresponding to 0.1 log unit change in stimuli luminance. Individual test loci threshold values are often termed *pointwise* *sensitivity*, and an average of these pointwise sensitivities provides the *mean sensitivity*, both of which are included as part of the standard S-MAIA output. A hill of vision volumetric spatial sensitivity, which we term *volume sensitivity* (measured in dB*deg^2^) was generated post hoc for each examination using the pointwise sensitivity values and loci coordinates, which can be exported from the machine as a text file and run through the custom scotopic MAIA volume app (ocular.shinyapps.io/scotopicMAIA).^27^

#### Structure–Function Analyses

Structure–function correlation analyses were undertaken using the measured areas of residual central retinal islands from the choroideremia cohort, which were obtained using fundus autofluorescence imaging, and corresponding microperimetry sensitivity data. These island areas were used as structural indicators of disease severity.^3^ The greater the island area of preservation, the less advanced the patient is in the course of the disease.

### Statistical Analyses

Descriptive and non-parametric analyses were performed using SPSS Statistics 29.0 (IBM, Chicago, IL), including medians and interquartile ranges (IQRs). Graphical figures were created using Prism 9.4.0 (GraphPad Software, Boston, MA). Pointwise variability was assessed via Bland–Altman analyses using a linear mixed-effects model to account for repeated measures and bootstrapping to estimate fixed-effect confidence intervals.^28^ Taking multiple measures into account where repeat measures are taken per participant (such as in pointwise sensitivities) is essential to avoid underestimating variability by considering the incorrect variances. Here, variability was quantified using the coefficient of repeatability (CoR) and is calculated using the usual formula:

$$\begin{array}{c} CoR=\;\sqrt{2}\;\times \;1.96\;\times SD_{within} \end{array}$$

However, it is important to note that *SD_within_* is the within-participant standard deviation obtained from the linear mixed-effects model rather than the overall standard deviation for all points across all participants. Tools for performing repeated-measures Bland–Altman analyses have been made freely available as the R package blandultim and can be installed from https://github.com/amanasj. This code was originally developed by Parker et al.^29^ and has been modified for use in ocular studies resulting in the blandultim R package. Cyan–red pointwise variability (after excluding test points with <0.0 dB at either Test 1 or Test 2) was also assessed using a linear mixed-model repeated-measures Bland–Altman analysis. Standard Bland–Altman analyses were used to assess test–retest variability of mean and volume sensitivities.^28^ The value for the CoR was used to identify the cohort representative level of natural variability and hence the minimum sensitivity changes required beyond natural variability to be considered clinically meaningful. As such, throughout we refer to this as *clinical significance*, separate from the requirements of statistical significance.

## Results

### Participant Demographics and Clinical Characteristics

**Figure 1.**
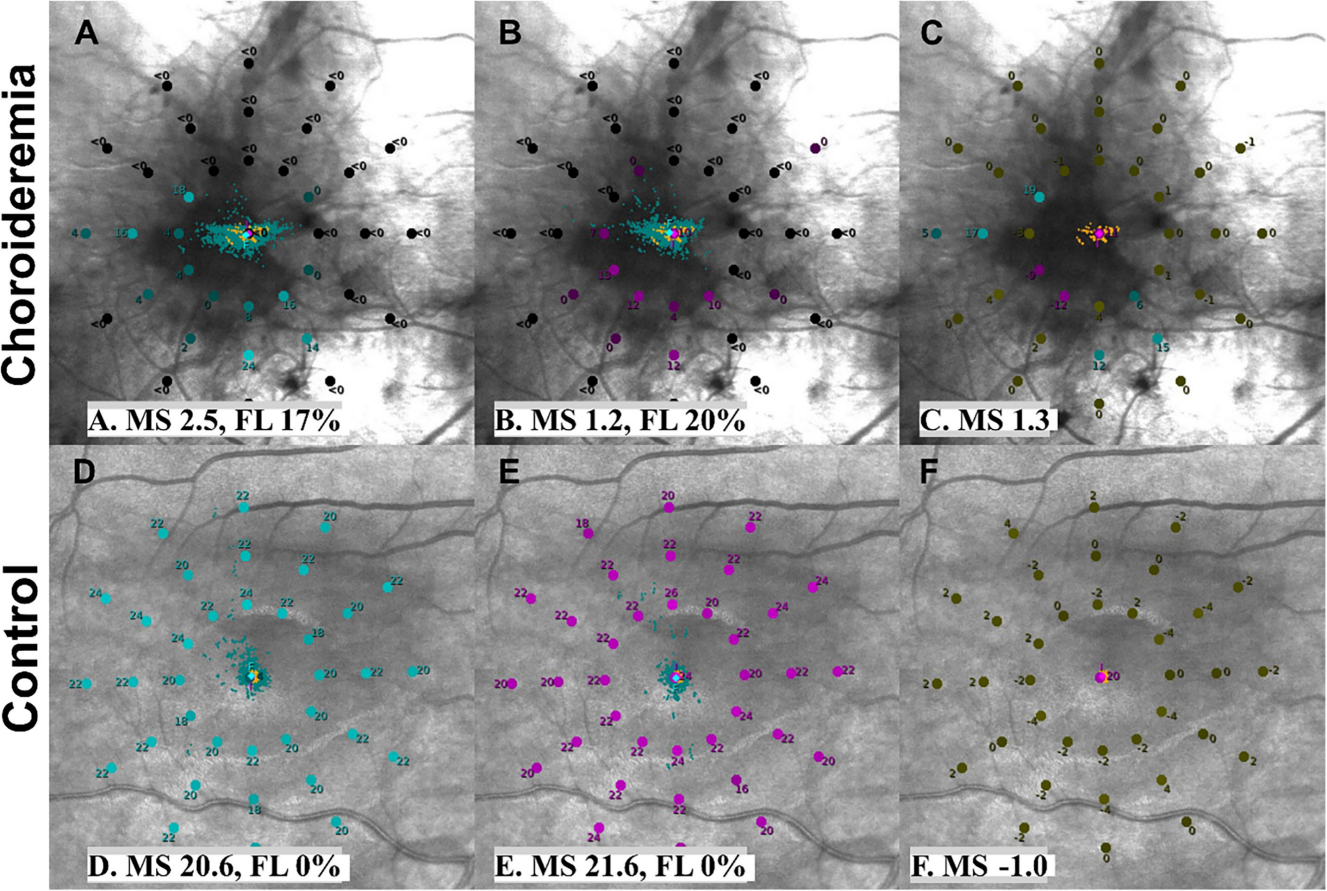
Pointwise scotopic microperimetry outputs including standard mean sensitivity (MS) and fixation loss (FL) indices. (**A**–**C**) The *top row* details the cyan, red, and cyan–red difference outputs for a participant with choroideremia. This participant had many tested loci with undetectable sensitivities, indicated by *black test points* (<0.0 dB). Four cyan-colored loci depicting greater cyan than red sensitivity suggest red (cone) dysfunction, whereas the three *red* *points* indicate reduced cyan sensitivity relative to red sensitivity, suggested cyan (rod) dysfunction. The *olive-green points* indicate equal cyan–red differences (within ±4.0 dB). (**D**–**F**) The *lower row* details cyan, red, and cyan–red difference pointwise sensitivity outputs for a healthy control participant. All points have equal differences (within ±4.0 dB), as indicated by the *olive-green* *points*, with only the central point showing reduced cyan sensitivity versus red sensitivity, which is characteristic of the rod-free fovea.

Twenty male patients with choroideremia (median age, 32 years; IQR, 21–45) completed scotopic microperimetry testing (with both cyan and red stimuli) in one eye only (Fig. 1). Thirteen participants completed testing with their right eye, and seven participants completed testing with their left eye, as a right-eye examination was contraindicated due to a history of previous ocular surgery or presence of significant visual impairment making testing unfeasible. In addition, 10 choroideremia patients completed repeat scotopic testing, and 11 also completed mesopic microperimetry testing on the same study eye for repeatability analyses. Eighty-four microperimetry tests from the choroideremia cohort were completed in total, 16 mesopic and 68 scotopic tests, as the cyan and red stimuli tests were undertaken separately. The median VA was 82 Early Treatment Diabetic Retinopathy Study (ETDRS) letters (IQR, 80–84).

Twenty-one age-matched healthy controls (nine males and 12 females; median age, 28 years; IQR, 24–42) also completed scotopic microperimetry testing (with both cyan and red stimuli) in one eye only (Fig. 1). Forty-two microperimetry tests in total were completed by healthy controls. Median VA was 91 ETDRS letters (IQR, 88–94). Although VA was significantly greater in controls compared to the choroideremia group (*P* < 0.001, Mann–Whitney *U* test), VA in the choroideremia group was considered relatively well preserved (≥6/9).

### Reliability Metrics

#### Fixation Losses

Zero-percent fixation losses were achieved in at least 60% of scotopic tests in both test groups, with cyan and red stimuli, from initial and repeat tests (Table 1). Similarly, the frequency of fixation losses was comparable with mesopic microperimetry testing (choroideremia cohort only). All of those with fixation losses >30% were excluded.

**Table 1.** Summary Statistics for the Microperimetry Reliability Data

	Choroideremia					Healthy Control	
	Scotopic Microperimetry Test 1 (*n* = 20)		Scotopic Microperimetry Test 2 (*n* = 14)		Mesopic Microperimetry (*n* = 16)	Scotopic Microperimetry (*n* = 21)	
	Cyan	Red	Cyan	Red	White	Cyan	Red
Reliability Indices							
Tests with no fixation losses, *n* (%)	13(65)	12 (60)	10 (71)	10 (71)	12 (75)	14 (67)	16 (76)
Tests with 0% < fixation losses < 30%, *n* (%)	4 (20)	7 (35)	4 (29)	4 (29)	3 (19)	5 (24)	3 (14)
Tests with high fixation losses ≥ 30%, *n* (%)	3 (15)	1 (5)	0 (0)	0 (0)	1 (6)	2 (10)	2 (10)
95% BCEA (deg^2^), median (IQR)	1.2 (0.6–3.4)	1.0 (0.5–1.8)	1.4 (0.6–3.4)	1.0 (0.6–2.2)	0.7 (0.4–1.3)	1.1 (0.5–2.5)	1.3 (0.8–2.4)
63% BCEA (deg^2^), median (IQR)	0.4 (0.2–1.1)	0.4 (0.2–0.8)	0.5 (0.2–1.1)	0.4 (0.2–0.7)	0.2 (0.1–0.7)	0.4 (0.2–0.8)	0.4 (0.2–1.2)
P1 (%), median (IQR)	99 (93–100)	98 (96–100)	96 (93–100)	98 (96–100)	99 (98–100)	98 (93–100)	98 (94–100)
P2 (%), median (IQR)	100 (99–100)	100 (100–100)	100 (98–100)	100 (100–100)	100 (100–100)	100 (100–100)	100 (100–100)
Stable fixation, *n* (%)	19/20 (95)	20/20 (100)	13/14 (93)	14/14 (100)	16/16 (100)	19 (100)	19 (100)
Central rod-free zone detected, *n* (%)	17/20 (85)		13/14 (93)		—	17/21 (81)	
Other Test Parameters							
Fixation target intensity, median (IQR)	35 (20–76)	44 (16–76)	60 (18–81)	44 (16–81)	—	5 (5–5)	5 (5–5)
Test duration (min:s), median (IQR)	5:08 (3:58–6.21)	5:10 (4:35–5:31)	4:37 (3:30–5:18)	4:39 (3:37–5:28)	8:03 (5:22–9.18)	5:17 (5.00–5:40)	4.55 (4:43–5:19)
Total testing duration (min:s), median (IQR)	10:33 (8:36–11:24)		9:54 (7:04–10:29)		—	10.10 (9.47–10.54)	

#### Fixation Stability

Cyan and red 95% and 63% BCEA values in the choroideremia group were generally very low (Table 1) and comparable with healthy controls and the mesopic BCEA values. This concordance indicates that this cohort of choroideremia patients, with well-preserved foveal function, could fixate effectively on the dim 1° red circular fixation target during scotopic testing. Overall, the majority of tests from both participant groups showed stable fixation when considering all of the fixation metrics analyses in Table 1. The fixation target intensity for participants with choroideremia was consistently required to be higher than that of healthy controls. The same fixation target intensity was used between Test 1 and Test 2, apart from in one individual who completed red Test 1 at a slightly lower fixation brightness than Test 2 (61.0 and 76.0 log units, respectively). The disparity in fixation intensity medians between scotopic Test 1 and Test 2, detailed in Table 1, is due to the reduced participant sample who completed repeat testing. The healthy control participants were all able to complete every test at the minimum fixation target intensity (5.0 log units).

#### Rod-Free Zone Mapping

The results for 85% of choroideremia Test 1 participants and 81% of healthy controls showed accurate detection of the central rod-free zone. Of the repeated choroideremia tests, 93% showed accurate mapping of the central rod-free zone.

The median central point cyan sensitivity was significantly lower in the choroideremia group than that of healthy controls (*P* = 0.008, Mann–Whitney *U* test). In choroideremia, the median central point cyan sensitivity was 0.1 dB (IQR, 0.0–2.5). Three choroideremia participants exhibited significantly higher central cyan point sensitivities (24.0 dB, 22.0 dB, and 12.0 dB), suggesting that these participants had unreliable rod-free zone mapping and they were excluded from further analyses. The median central point cyan sensitivity in healthy controls was 4.0 dB (IQR, 3.0–8.0). Four healthy control participants had central point cyan sensitivity greater than 8.0 dB, which tentatively also suggests an absence of rod-free zone mapping and poor reliability. These four were also excluded from further analyses.

#### Excluded Tests

Following a review of all reliability measures, 11 tests (9%) were excluded. These included seven tests from seven different participants with choroideremia from the Test 1 phase and four tests from four different healthy control participants. A further two tests, from two different participants with choroideremia, were deemed unreliable in the repeat testing. Interestingly, these tests corresponded to participants who also had their initial tests excluded and so overall were deemed unreliable responders. Supplementary Table S1 lists the excluded participants, with details in blue highlighting the reason for exclusion. High fixation losses and the absence of the rod-free zone mapping were the main causes of exclusion in both healthy controls and choroideremia participants. There was no obvious trend between cyan and red testing and poor reliability, despite cyan testing being performed first, suggesting no obvious learning effects. Two choroideremia participants with excluded tests had repeat testing that was deemed reliable. These repeat tests were included for subsequent analyses.

### Central Macular Sensitivity Analyses

#### Pointwise Analyses

**Figure 2.**
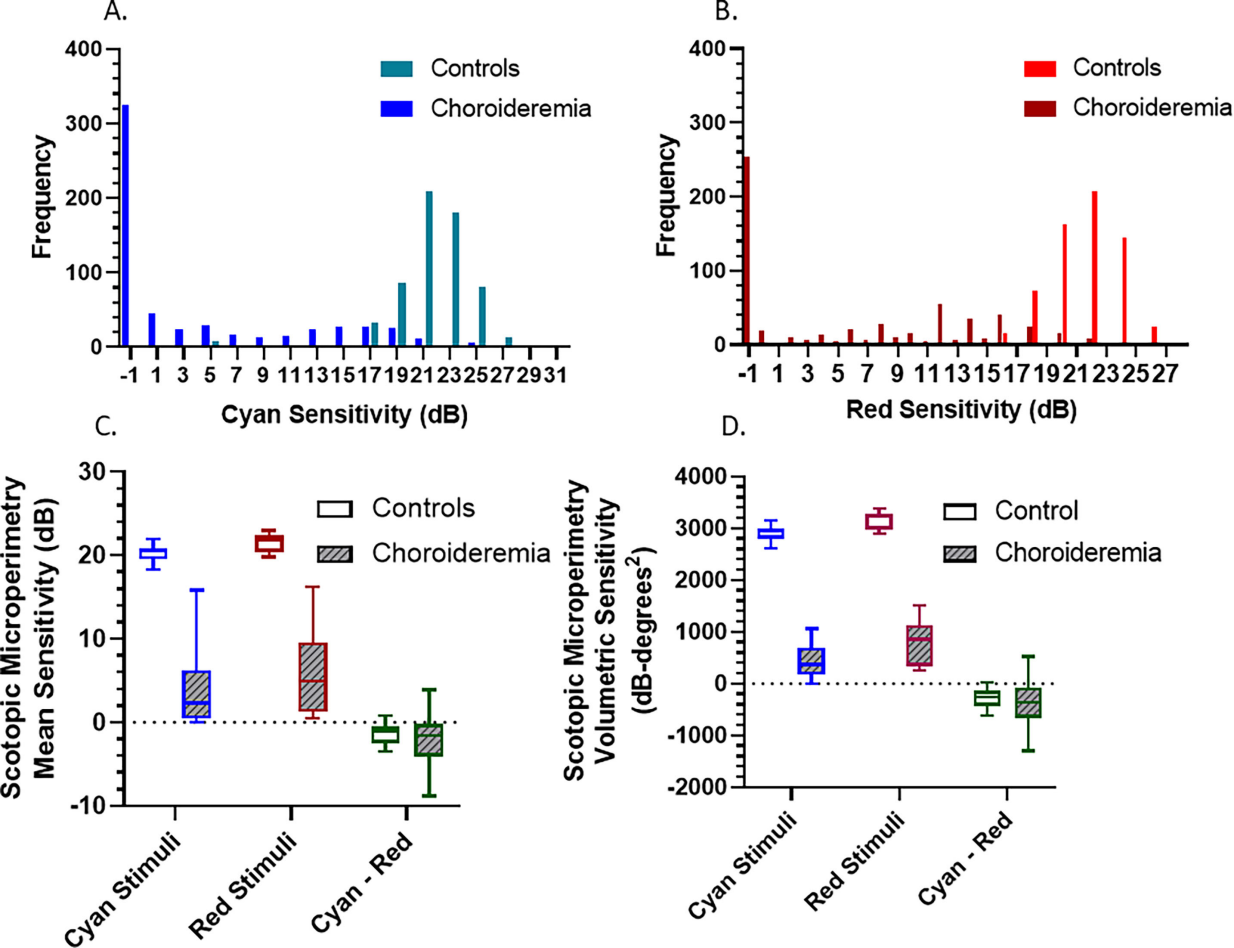
(**A**) Frequency distribution of pointwise sensitivity values for cyan stimuli. (**B**) Frequency distribution of pointwise sensitivity values for red stimuli. (**C**, **D**) Shown are the cyan and red mean sensitivity (**C**) and volume sensitivity (**D**) values for both healthy controls (*n* = 17) and choroideremia participants (*n* = 16), as well as the cyan–red volume difference.

In choroideremia, 592 threshold loci (37 test points × 16 participants) were tested for cyan and red stimuli testing. Both cyan and red stimuli showed a skewed distribution toward –1.0 dB (Figs. 2A, 2B), and the remaining loci demonstrated generally low decibel threshold sensitivity values. The skewed distribution is due to large floor effects, where 325 cyan loci and 254 red loci had no detectable sensitivity and are arbitrarily assigned –1.0 dB. With a larger dynamic range, it would be expected that the distribution would normalize, albeit at a lower central peak than that for healthy controls. In comparison, healthy controls showed normally distributed sensitivity values from cyan and red testing on 629 threshold loci (37 test loci × 17 participants) (Fig. 2). Only a small number of points from cyan testing had <10.0 dB corresponding to the central fovea locus.

#### Mean Sensitivity

Cyan and red mean sensitivities were significantly reduced in the choroideremia group compared to those of the healthy controls (P < 0.001, Mann–Whitney *U* test). All healthy controls had measurable mean cyan and red sensitivities, with median mean sensitivities of 20.6 dB (IQR, 19.6–20.9) for cyan and 21.8 dB (IQR, 20.4–22.4) for red. In choroideremia, the median mean sensitivities were 2.3 dB (IQR, 0.4–6.3) for cyan and 4.9 dB (IQR, 1.3–10.0) for red. Three participants had 0.0-dB cyan mean sensitivity, whereas three others had very low values, defined as <1.0 dB cyan mean sensitivity, due to large numbers of loci with no detectable sensitivity and all loci results being included in the mean sensitivity output. All participants with choroideremia revealed detectable sensitivity with red stimuli, and only two participants had very low (<1.0 dB) red mean sensitivity. Compound sensitivity heatmaps showed statistically greater temporal mean sensitivity than nasal mean sensitivity for both cyan (temporal, 5.5 dB; nasal, 3.2 dB; *P* < 0.001) and red stimuli (temporal, 7.7 dB; nasal, 4.8 dB; *P* < 0.001) (Supplementary Fig. S1).

#### Volume Analyses

Median cyan and red volume sensitivity was statistically significantly lower in choroideremia compared to healthy controls (*P* ≤ 0.001, Mann–Whitney *U* test) (Fig. 2). In healthy controls, the median volume sensitivities were 2953.8 dB*deg^2^ (IQR, 2880.9–2988.5) for cyan and 3230.1 dB*deg^2^ (IQR, 2984.0–3286.2) for red. In choroideremia, the median volume sensitivities were 462.2 dB*deg^2^ (IQR, 178.6–998.3) for cyan and 878.0 dB*deg^2^ (IQR, 366.4–1502.5) for red.

All participants, in both groups, showed measurable volume sensitivity; two participants with choroideremia had very low cyan volume sensitivity of 0.9 dB*deg^2^ (0.0-dB mean sensitivity) and 0.5 dB*deg^2^ (0.0-dB mean sensitivity). Volume sensitivity values were unaffected by the averaging floor effects experienced with mean sensitivity.

**Figure 3.**
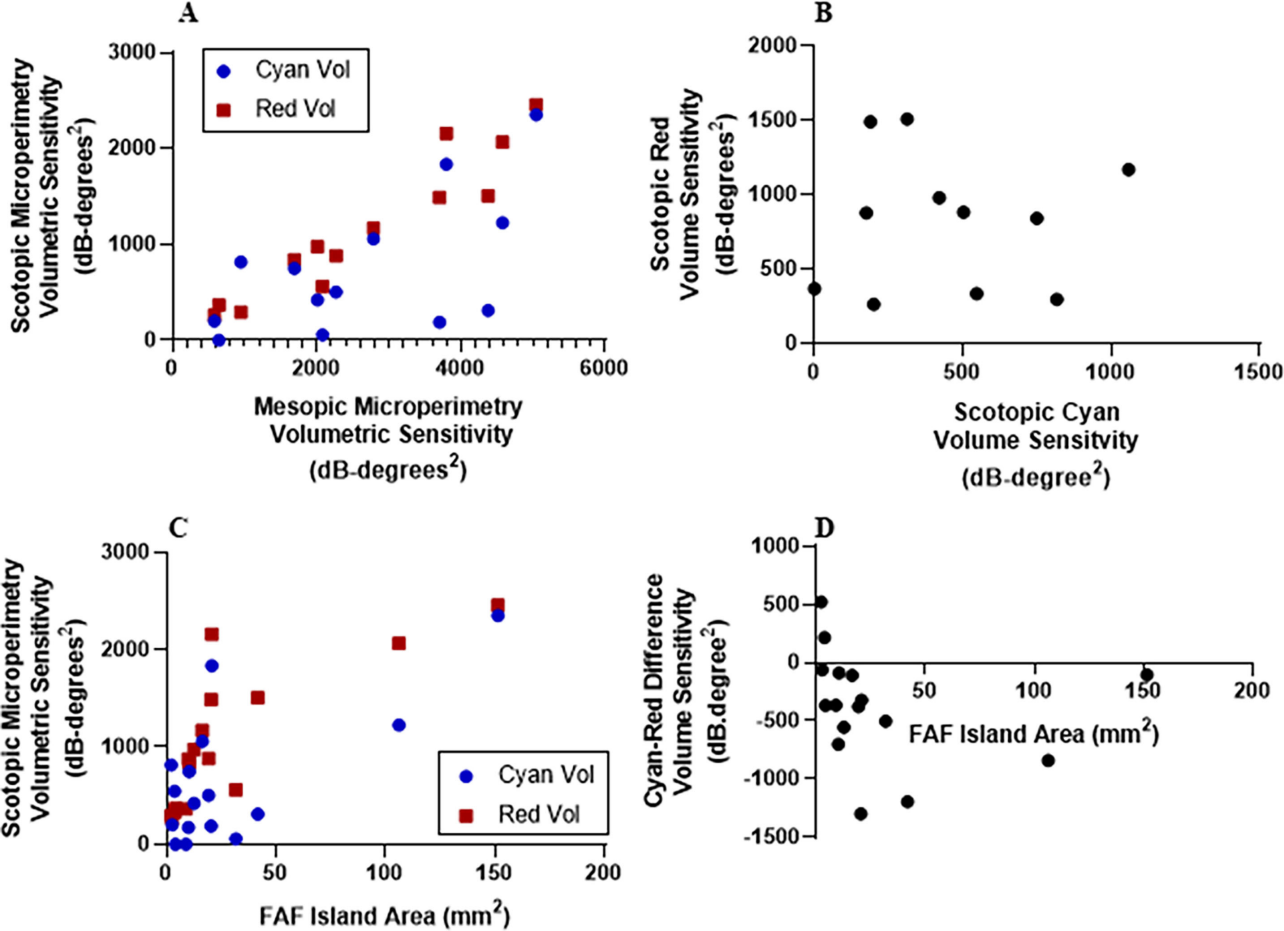
Scotopic microperimetry correlation analyses in choroideremia. (**A**) Mesopic microperimetry volumetric sensitivity correlated significantly with cyan volume sensitivity (ρ = 0.56; *P* = 0.05) and red volume sensitivity (ρ = 0.95; *P* < 0.01). (**B**) There was no correlation between cyan volumetric sensitivity and red volumetric sensitivity (ρ = 0.26; *P* = 0.30). (**C**) The areas of preserved seeing islands identified with fundus autofluorescence in choroideremia correlated significantly with red volume sensitivity (ρ = 0.89; *P* < 0.01) but not with cyan volume sensitivity (ρ = 0.36; *P* = 0.17). (**D**) Cyan–red difference correlated significantly (ρ = −0.56; *P* = 0.02).

There was no significant correlation between age and cyan or red volume sensitivity, indicating either an insufficient sample size or reflective of the heterogeneity of phenotypes across different ages with this cohort (Supplementary Fig. S2). Mesopic microperimetry volume sensitivity correlated significantly with cyan volume sensitivity and showed an even stronger significant correlation with red volume sensitivity (Fig. 3A). This reinforces the premise that mesopic and scotopic red microperimetry sensitivities are reflective of associated retinal cone function, whereas scotopic cyan sensitivities are potentially partly reflective of an alternative retinal function—namely, rod function.

#### Cyan–Red Difference

A unique feature of S-MAIA two-color perimetry is the ability to spatially compare the pointwise cyan and red thresholds, which allows us to understand the relative differences and identify localized specific photoreceptor function and dysfunction (Table 2; Figs. 1C, 1F). Cyan mean and volume sensitivities were statistically lower than the red mean and volume sensitivities in both healthy controls and choroideremia (Table 3). Spearman rank correlation analyses revealed no significant correlation between cyan and red mean and volume sensitivities in healthy controls (ρ = 0.28, *P* = 0.29; ρ = 0.26, *P* = 0.30, respectively), but they did reveal a statistically significant correlation between cyan and red mean and volume sensitivities in choroideremia (ρ = 0.58, *P* = 0.02; ρ = 0.52, *P* = 0.04, respectively) (Fig. 3B).

**Table 2.** Tool to Aid in the Interpretation of Pointwise Difference Plots

Cyan–Red (dB)	MAIA Color Code	Cyan Stimuli Sensitivity	Red Stimuli Sensitivity	Interpretation	Remodeled Color Code
0	Olive-green	Normal	Normal	Normal dark-adapted function	Gray
0	Olive-green	Reduced	Reduced	Equally impaired cone and rod function	Black
<0	Red	Reduced	Normal or mildly reduced	Impaired rod function relative to cone function	Red
>0	Cyan	Normal or mildly reduced	Reduced	Impaired cone function relative to rod function	Blue

Adapted from Heeren et al.^15^

**Table 3.** Cyan–Red Summary Statistics

	Heathy Controls	Choroideremia
Mean sensitivity		
Cyan–red (dB), median (IQR)	−1.1 (−2.5 to −0.5)	−1.6 (−1.4 to −0.2)
*P*, cyan versus red	−0.01	0.02
Volume sensitivity		
Cyan–red (dB*deg^2^), median (IQR)	−264.0.6 (−430.0 to −219.8)	−366.2 (−92.9 to −366.2)
*P*, cyan versus red	0.002	0.007

### Test–Retest Variability

Ten choroideremia participants completed repeat testing, each repeating all 37 test points for both cyan and red stimuli. This resulted in 370 test loci for each stimuli. The measured sensitivity values between tests one and two were the same in 158/370 test loci (43%) and 192/370 test loci (52%) for cyan and red stimuli, respectively, including test points with no detectable sensitivity. Furthermore, 218/370 loci (59%) were within a range of –2.0 to 2.0 dB for cyan stimuli testing, and 290/370 loci (78%) were within a range of –2.0 to 2.0 dB for red stimuli testing. Table 4 and Supplementary Figure S3 detail the CoR for pointwise sensitivity after accounting for repeated measures, mean sensitivity, and volume sensitivity. Cyan stimuli showed greater variability than red stimuli across all three indices, as indicated by the higher coefficients of repeatability. Despite this, only red volume sensitivity showed a statistically significant difference between Test 1 and Test 2. Because scotopic red repeat testing was performed last, this could be suggestive of fatigue effects.

**Table 4.** Test–Retest Variability Data for Pointwise, Mean, and Volume Cyan and Red Sensitivities

	Cyan Stimuli	Red Stimuli
Pointwise sensitivity		
CoR (dB)	±15.5	±12.4
Mean sensitivity		
Test 1 (dB), median (IQR)	2.6 (1.5–6.3)	4.9 (1.4–10.6)
Test 2 (dB), median (IQR)	2.1 (0.6–6.3)	5.2 (1.3–10.3)
Significance (Wilcoxon signed-rank test)	*P* > 0.05	*P* > 0.05
CoR (dB)	±3.3	±1.4
Volume sensitivity		
Test 1 (dB*deg^2^), median (IQR)	525.3 (278.1–525.3)	878 (357.0–1669.6)
Test 2 (dB*deg^2^), median (IQR)	427.4 (221.2–1050.1)	714.9 (265.1–1596.0)
Significance (Wilcoxon signed-rank test)	*P* > 0.05	*P* = 0.04*
COR (dB*deg^2^)	±527	±217

#### Cyan–Red Pointwise Difference

**Figure 4.**
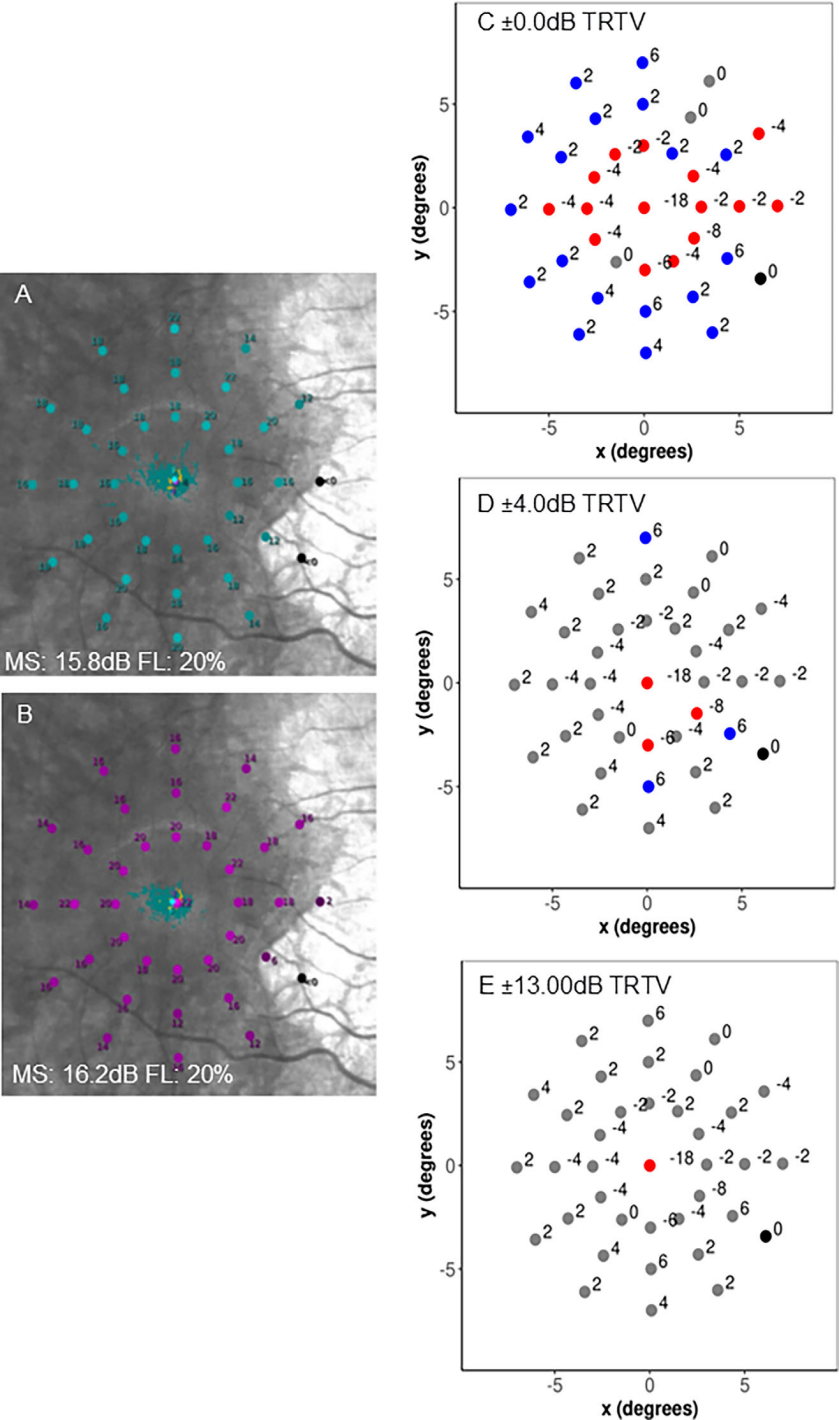
The variation in cyan–red difference plots for a single patient after accounting for different degrees of pointwise test–retest variability (TRTV). Standard output cyan pointwise plot (**A**) and red pointwise plot (**B**), with mean sensitivity (MS) and fixation loss (FL) indices noted, for a single choroideremia participant with well-preserved central visual function. (**C**–**E**) Remodeled cyan–red difference pointwise plots after accounting for different degrees of test–retest variability. *Gray* *points* lie within the set test–retest variability, *blue points* indicate cyan dysfunction, and *red points* indicate red dysfunction. Cyan–red differences are shown with no test–retest variability accounted for (**C**). Cyan versus red dysfunctional differences are highlighted after accounting for ±4.0-dB variation, which corresponds to the arbitrary cut-off used by the S-MAIA difference output plot (**D**). The cyan–red difference plot was remodeled to account for the ±13.0-dB combined pointwise test–retest variability (**E**).

For accurate interpretation of cyan versus red sensitivity, it is important to consider the combined test–retest variability. Currently, the S-MAIA cyan–red difference plot uses an arbitrary ±4.0-dB limit to highlight potentially dysfunctional cyan versus red relative differences (Figs. 1C, 1F). The combined pointwise CoR for cyan–red difference (excluding 0.0-dB values) was ±12.8 dB (Supplementary Fig. S3C), which we have rounded to ±13.00 dB for simplicity. This suggests that a difference of >13.0 dB is required to identify a clinically meaningful cyan–red difference value as being attributable to a relative rod versus cone dysfunction. This clearly limits the ability to detect subtle levels of relative pointwise dysfunction, as illustrated by Figure 4.

#### Cyan–Red Mean and Volume Sensitivities

Bland–Altman test–retest analyses from the subcohort of participants with choroideremia who completed the repeat testing (reported above) showed a combined cyan–red mean sensitivity CoR of ±2.7 dB. This suggests that, for a clinically significant sensitivity difference between the two stimuli that is beyond natural variability, a cyan–red mean sensitivity difference > 2.7 dB is required. The combined cyan–red volume sensitivity CoR was ±413.8 dB*deg^2^ for choroideremia, suggesting that a cyan–red difference of >413.8 dB*deg^2^ is required for a clinically meaningful difference beyond natural variability.

### Structure–Function Association

The median island area was 14.6 mm^2^ (IQR, 5.5–29.0). The residual area correlated significantly with red volume sensitivity (ρ = 0.89; *P* < 0.01, Spearman rank) (Fig. 3C), suggesting that, as the disease progresses, scotopic red sensitivity is reduced. However, there was no correlation with cyan sensitivity (ρ = 0.36; *P* = 0.17, Spearman rank) (Fig. 3C), suggesting that cyan sensitivity declines independently of visible photoreceptor degeneration. However, the cyan–red difference correlated significantly with residual fundus autofluorescence island areas (ρ = –0.56; *P* = 0.02, Spearman rank) (Fig. 3D), highlighting that in earlier disease stages (indicated by larger central islands) there are larger negative cyan–red volume sensitivity differences. This suggests the presence of greater cyan dysfunction (rod dysfunction) in early disease stages compared with red dysfunction (cone dysfunction). With further disease progression, the residual island area reduces, along with the cyan–red difference, likely due to the onset of impaired cone (red) function, after earlier rod (cyan) impairment. This highlights the benefits of interpreting the cyan–red difference relative to individual red and cyan sensitivities, and could have significant implications for the detection and monitoring of early disease.

On the other hand, Supplementary Figure S4 details scotopic sensitivity plots from three example choroideremia participants, overlayed onto their corresponding fundus autofluorescence images. Because many testing points fall outside the residual seeing area, the figure illustrates the limitations of using the default 37-point radial scotopic grid in patients with choroideremia.

## Discussion

This study explored the suitability of scotopic microperimetry as a clinical trial outcome measure for choroideremia through a rigorous analysis of summary statistics and reliability metrics. All participants (patients with choroideremia and healthy controls) were able to complete testing. Scotopic microperimetry sensitivity was reduced in participants with choroideremia, compared to healthy controls, whereas VA remained relatively well preserved, suggesting that scotopic microperimetry is superior to detecting earlier visual function changes than VA alone. This could be of significant benefit for earlier diagnosis and for clinical trials investigating potential novel therapies. However, the test–retest variabilities in scotopic microperimetry were higher than those previously reported for mesopic microperimetry in choroideremia (±1.5 dB mean sensitivity and ±4.7 dB central pointwise sensitivity).^4^^,^^5^ Alongside the high number of tests excluded due to poor reliability, this finding may limit the potential of scotopic microperimetry as a robust outcome measure.

Fixation stability did not appear to be a causative factor in poor test reliability. Participants with choroideremia were able to maintain stable fixation with low BCEA scores comparable to those of healthy controls.^30^ The S-MAIA does not officially provide false-positive catch trials within the testing algorithm, thus making it difficult to detect unreliable responders. High fixation losses were the leading cause of test exclusions, corresponding to responses to stimuli presented to the physiological blind spot. In the presence of stable fixation, arguably these responses could be considered false positives, arising from unintentional button presses, which could indicate participant difficulties with the testing task (i.e., threshold stimulus detection in scotopic conditions).

The physiological absence of rod photoreceptors at the fovea means that, in theory, patients should not have any cyan sensitivity (under scotopic conditions) at the central fovea testing loci.^31^ Therefore, absence of the rod-free zone was used as a novel reliability marker and, in the presence of stable fixation, as a proxy for false-positive test responses. This approach assumed alignment between the anatomical and physiological fovea and correct positioning of the grid center on the fovea. The detection of the rod-free zone was deemed successful as an additional reliability marker and was the second highest cause of test exclusions in the choroideremia group. However, when using the default scotopic 37-point radial grid, as used in this study, there is only one presentation within the rod-free zone at the foveal center. This limits its use somewhat, as a single rapid saccade (undetected by the S-MAIA 25-Hz eye-tracking software) may enable viewing of the central loci via a more sensitive parafoveal retinal location,^30^ or simply an unintended button press may lead to an erroneous result. Having several central loci (within the fovea region) may make rod-free zone mapping a more useful reliability indicator in a manner similar to blind-spot testing. However, because the size of the rod-free zone is variable from person to person, it may be difficult to determine appropriate spacing of multiple central loci.^32^ Repeat threshold sensitivity testing of central loci during a single examination may be more practical. Alternatively, incorporation of more formal false-positive and false-negative catch trials, similar to those used in standard static perimetry devices, may improve assessments of reliability, although with the caveat that examination times are often increased as a result.

The rate of wrong pressure events has previously been suggested by Montesano et al.^23^ to be a robust reliability marker. However, this requires careful post-assessment analyses of the response time data for each stimuli and identification of button presses occurring outside this window, which is currently not a standard output on the S-MAIA device.^18^^,^^23^ In a clinical setting, clinicians require readily available reliability indicators to determine whether a test is reliable or whether it needs repeating; hence, wrong pressure events analyses were not used in this study.

Identification of the most appropriate scotopic microperimetry sensitivity index is another important consideration. Pointwise indices showed large test–retest variability. Mean sensitivity demonstrated smaller test–retest variability, as large localized (pointwise) changes are averaged. Mean sensitivity is also easy to interpret and readily available on MAIA outputs. However, mean sensitivity does not accurately represent the overall sensitivity, particularly in those with many non-seen points (due to very constricted central visual fields), where the distribution of pointwise sensitivities is heavily skewed toward 0.0 dB. Furthermore, the radial testing grid used leads to a spatially weighted mean result.^33^ A rectilinear grid, such as the 68 point 10-2, would eliminate spatially weighted averaging; however, this would result in an unacceptable increase in testing times. Therefore, the suitability of mean sensitivity in scotopic microperimetry in patients with choroideremia is very limited. Conversely, volume sensitivity is a product of sensitivities and spatial locations; it is not affected by averaging effects of non-seen loci and is immune to any biases associated with sampling densities and associated weighted averaging. This results in a greater range of lower sensitivity values in those with late-stage disease.^32^^,^^33^ To the best of our knowledge, this is the first time that volume sensitivity analyses have been used for scotopic microperimetry testing in patients with choroideremia.

In choroideremia, greater retinal sparing is typically seen on the temporal macular area.^4^ For optimal test grid design, loci density should perhaps be more concentrated on the temporal region. Alternatively, with the advent of volume sensitivity indices, the use of custom microperimetry testing grids, tailored to each participant's residual preserved central seeing island, would enable the most effective and comparable sensitivity assessment.^33^^,^^34^

Only the S-MAIA microperimeter contains built-in two-color fundus-controlled microperimetry testing capability. Although other fundus-controlled microperimetry testing devices are available, such as the NIDEK MP-1 and NIDEK MP-3, these only provide scotopic testing with a single stimuli color, unless extra filters are included.^14^ Two-color scotopic microperimetry offers the potential to spatially assess cyan (preferentially rod function) relative to red (mixed cone–rod function). This is particularly appealing when investigating cases such as reduced rod function reported in age-related macular degeneration secondary to choroidal neovascularization.^18^ However, the high variability of pointwise sensitivities limits the usefulness of the pointwise cyan–red difference analyses and, hence, any inference of rod versus cone dysfunction in any given region. Global measures such as mean and volume sensitivities, which are less prone to variability, may still provide valid cyan–red relative comparisons to help develop an understanding of the underlying disease mechanisms. However, these are limited, as they include the central locus threshold sensitivity, which falls within the rod-free zone. As a result, all global cyan–red differences are skewed toward indicating cyan dysfunction, as seen in healthy controls. Exclusion (post hoc or by grid design) of the central locus from global analyses is necessary to eliminate this bias.

In patients with rod–cone degenerations, with a resulting absence or severe reduction of rod function, it is likely that cones are able to detect cyan stimuli at the brighter stimulus levels.^35^ The average sensitivity of the central cyan point (at the center of the rod-free zone) in healthy controls was found to be above zero and contrary to expectation. This may be suggestive of cone photoreceptor response at the brightest cyan levels. This limits the assumption that all cyan sensitivity responses are elicited solely from rod photoreceptors, particularly in patients with rod-cone degeneration. Once cyan sensitivity becomes very low, as seen in choroideremia (Fig. 2A), our findings suggest that any responses could be reflective of cone responses. Furthermore, the significant correlation between cyan and red mean and volume sensitivities in choroideremia (but not in healthy controls) suggests that either rod function is so impaired that cones are responding to both cyan and red stimuli, or both rod and cone photoreceptors are degenerating in a correlated fashion. Overall, there appears to be significant ambiguity in the interpretation of cyan–red differences. In addition, the very large test–retest repeatability coefficient associated with the cyan–red index suggests that this output, given as part of the standard output, is unlikely to be a useful outcome measure for use in clinical trials.

Study limitations include no formal learning test being undertaken, as most participants were experienced in microperimetry and we wanted to minimize test time and subsequent fatigue. Only those who were completely microperimetry naïve underwent a short eight-point cyan threshold exam to ensure understanding. Many participants were familiar with the core microperimetry task, which involves maintaining central fixation and pressing the button in response to stimuli. A separate learning component involves the ability to accurately respond to stimuli at threshold levels, which is difficult, particularly in scotopic conditions, and could be a source of variability that might be reduced if every participant completed a short threshold training test prior to full testing. Despite this, overall, the repeat testing results were mostly comparable, suggesting no major learning effects. Only red volume sensitivity repeat results showed statistically significant lower sensitivities in choroideremia. Because red is tested after cyan, this is potentially more likely to reflect fatigue effects.

Another limitation is dark adaptation, as only 20 minutes of dark adaptation time was undertaken prior to testing, although this has been recommended by previous studies^21^^,^^22^ as it balances the time to reach adequate rod recovery and testing time practicalities. There were no additional checks undertaken to ensure that participants were adequately dark adapted. In choroideremia participants, dark adaptation has been shown to take longer than healthy controls^36^; therefore, it could be argued that perhaps patients with choroideremia may need longer time to ensure adequate cyan testing sensitivity and to reduce test variability.^37^ However, because every participant is different, standardizing the level of dark adaptation is difficult.

Sex was not controlled for, all participants with choroideremia were male, and the control group was mixed. However, retinal sensitivity and visual function has been shown to be similar for males and females.^38^^,^^39^ The study is also limited by the small sample size; due to the rarity of choroideremia, it is difficult to recruit large numbers of participants. Further investigation with younger earlier stage patients (under 16 years of age) would be beneficial to identify test suitability, particularly as scotopic microperimetry is a difficult test to perform, and it may be too tiring or difficult for younger patients.

## Conclusions

Scotopic microperimetry may be a useful measure of central retinal spatial scotopic sensitivity in patients with early choroideremia who still have well-preserved central retinal islands and can demonstrate that they can perform the tests reliably. Considering the testing time and scotopic testing environment requirements, scotopic microperimetry is unlikely to be suitable as a screening tool to aid early disease detection and diagnosis. However, scotopic microperimetry could be used to aid scotopic visual function monitoring and as an outcome measure in future therapeutic clinical trials. This is particularly relevant in those with early-stage disease, where other visual function measures remain preserved, which limits the potential for immediate measurable visual function improvements in clinical trials, and subsequent regulatory approval.

Careful consideration is necessary with regard to scotopic microperimetry test grid design, the provision of training tests, and the selection of sensitivity indices. Test–retest variability must be considered to determine the level of sensitivity change required for a clinically significant result. Caution should be exercised with the cyan–red difference index, as it suffers from poor repeatability, which limits its potential usefulness. In addition, use of the cyan–red differences to indicate relative rod or cone dysfunction is contraindicated due to the significant potential for mis-interpretation, particularly in the presence of severe rod dysfunction.

Further investigation is needed to characterize scotopic microperimetry in various retinal degenerations, especially in rod–cone and cone–rod dystrophies where it is unclear what process is dominating the response. Having a large dataset at different disease stages and corroborating with mesopic microperimetry and structural measures, such as fundus autofluorescence, may help develop an understanding of scotopic microperimetry and functional interpretation. Most inherited and acquired retinal diseases have now been well characterized using mesopic microperimetry and static automated perimetry, and it would also be beneficial to have every disease characterized using scotopic microperimetry.

## Supplementary Material

Supplement 1
