# Supplementary material for: Exploring Scotopic Microperimetry as an Outcome Measure in Choroideremia
Source: Transl Vis Sci Technol. 2024 Sep 30;13(9):29. doi: 10.1167/tvst.13.9.29 (PMC11441449; doi:10.1167/tvst.13.9.29)
Supplement: Supplement 1 [file tvst-13-9-29_s001.pdf]

Table S1: Scotopic microperimetry summary data for excluded tests.

Excluded px ID	Age	VA	Excluded Test Type	Fixation losses %	95BCEA	63BCEA	P1	P2	Fixation Stability	Rod free zone mapping
**Choroideremia**										
1	20	84	Cyan Stimuli	**33**	0.5	0.2	100	100	stable	yes
2	34	82	Red Stimuli	20	0.9	0.3	98	100	stable	**no**
3	31	81	Red Stimuli	0	1.8	0.6	95	100	stable	**no**
4	49	87	Red Stimuli	**40**	0.4	0.1	100	100	stable	yes
5	24	85	Cyan Stimuli	**33**	0.6	0.2	100	100	stable	**no**
6	17	75	Cyan Stimuli	**33**	7.6	2.5	93	95	stable	yes
7	27	80	Cyan Stimuli	0	52.5	8.6	4	22	**unstable**	yes
**Healthy Controls**										
1	39	90	Cyan Stimuli	**40**	4.8	1.6	91	97	stable	**no**
2	25	94	Cyan Stimuli	**60**	0.5	0.2	100	100	stable	**no**
3	20	91	Red Stimuli	**33**	0.5	0.2	99	100	stable	**no**
4	29	92	Red Stimuli	**33**	2.4	0.8	95	100	stable	yes

*Blue text highlights the reason for the test being excluded.

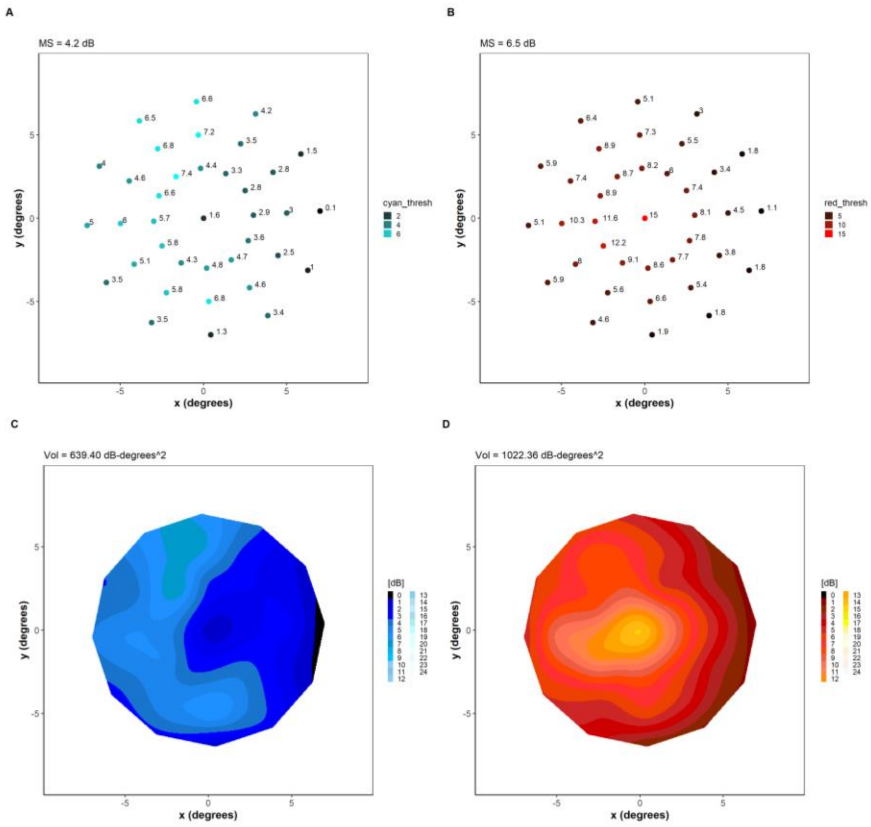

Figure S1: Compound mean pointwise sensitivity plots including the entire choroideremia cohort for scotopic cyan (A) and scotopic red (B). Compound heatmaps for scotopic cyan(C) and scotopic red sensitivity (D) indicating temporal versus nasal macular sensitivity asymmetry.

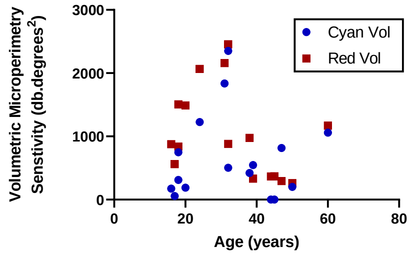

Figure S2: Scotopic microperimetry and age in choroideremia. There was no significant correlation between age and scotopic cyan (ρ=0.11, P=0.69) and red sensitivity (ρ=-0.41, P=0.11).

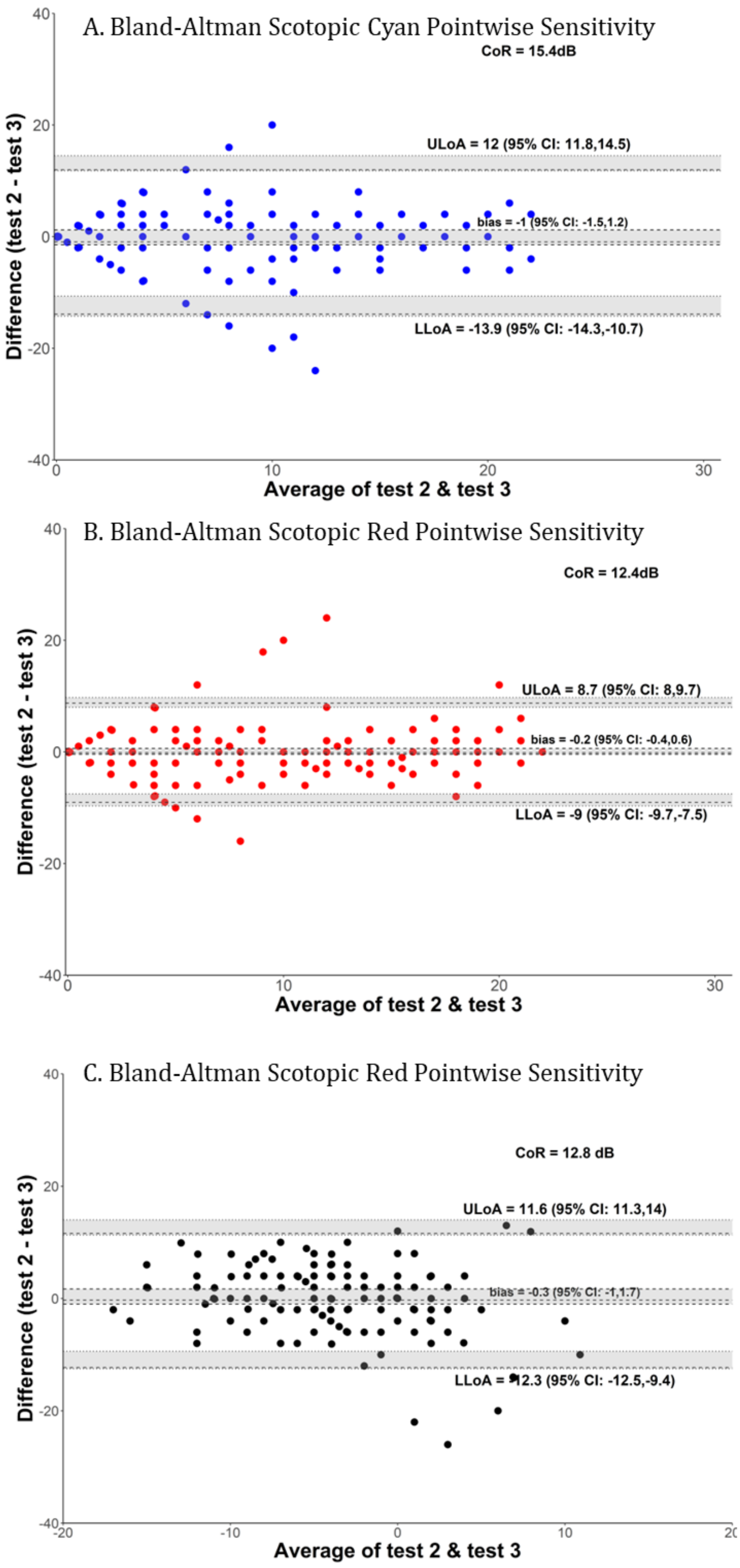

Figure S3: Bland-Altman analysis using Bayesian bootstrapping to account for repeated measures for scotopic cyan (A) and scotopic red (B) pointwise sensitivities from a cohort of patients with choroideremia (n=10). C represents the cyan-red difference combined Bland-Altman repeatability plot.

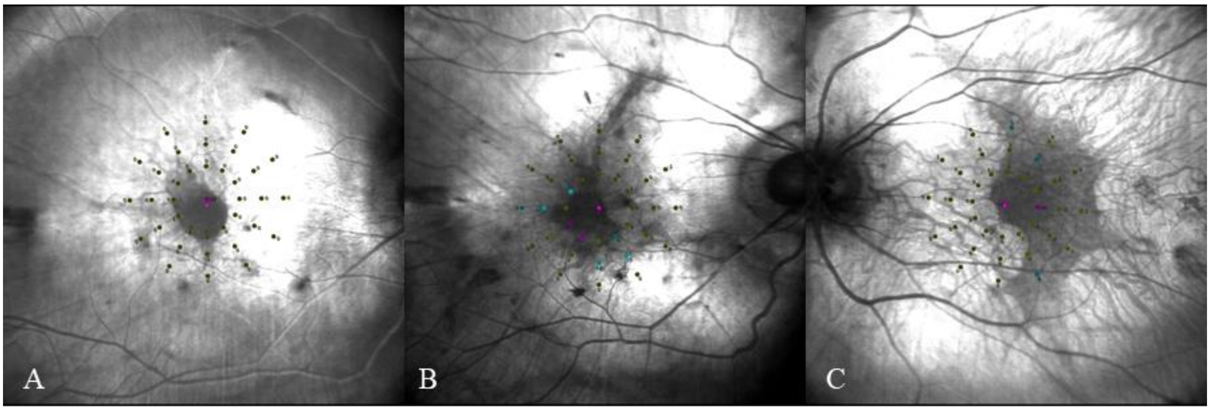

Figure S4: Scotopic cyan minus scotopic red difference plots overlayed on to fundus autofluorescence images from three participants with choroideremia indicating test grid unsuitability using imageJ (Version 1.53T and plugin: align image by line ROI V3 0.4). (A) Details how the central island is mostly unassessed as it is within the central ring of testing points. (B) Details another patient's residual island falls outside the testing grid. (C) Shows a patient with a larger preserved temporal island.
